# Construction and characterization of a full-length cDNA library for the wheat stripe rust pathogen (*Puccinia striiformis *f. sp. *tritici*)

**DOI:** 10.1186/1471-2164-8-145

**Published:** 2007-06-04

**Authors:** Peng Ling, Meinan Wang, Xianming Chen, Kimberly Garland Campbell

**Affiliations:** 1 US Department of Agriculture, Agricultural Research Service, Wheat Genetic, Quality, Physiology and Disease Research Unit, Pullman, WA 99164-6430, USA; 2 Department of Plant Pathology, Washington State University, Pullman, WA 99164-6430, USA; 3 College of Plant Protection, Northwest A&F University, Yangling, Shaanxi, People's Republic of China; 4 Department of Soil and Crop Sciences, Washington State University, Pullman, WA 99164-6420, USA

## Abstract

**Background:**

*Puccinia striiformis *is a plant pathogenic fungus causing stripe rust, one of the most important diseases on cereal crops and grasses worldwide. However, little is know about its genome and genes involved in the biology and pathogenicity of the pathogen. We initiated the functional genomic research of the fungus by constructing a full-length cDNA and determined functions of the first group of genes by sequence comparison of cDNA clones to genes reported in other fungi.

**Results:**

A full-length cDNA library, consisting of 42,240 clones with an average cDNA insert of 1.9 kb, was constructed using urediniospores of race PST-78 of *P. striiformis *f. sp. *tritici*. From 196 sequenced cDNA clones, we determined functions of 73 clones (37.2%). In addition, 36 clones (18.4%) had significant homology to hypothetical proteins, 37 clones (18.9%) had some homology to genes in other fungi, and the remaining 50 clones (25.5%) did not produce any hits. From the 73 clones with functions, we identified 51 different genes encoding protein products that are involved in amino acid metabolism, cell defense, cell cycle, cell signaling, cell structure and growth, energy cycle, lipid and nucleotide metabolism, protein modification, ribosomal protein complex, sugar metabolism, transcription factor, transport metabolism, and virulence/infection.

**Conclusion:**

The full-length cDNA library is useful in identifying functional genes of *P. striiformis*.

## Background

*Puccinia striiformis *Westend., a fungus in Pucciniacea, Uredinales, Basidiomycotina, Eumycota, causes stripe (yellow) rust. Based on specific pathogenicity on cereal crops and grasses, the fungal species consists of various formae speciales, such as *P. striiformis *f. sp. *tritici *on wheat (*Triticum aestivum*), *P. striiformis *f. sp. *hordei *on barley (*Hordeum vulgare*), *P. striiformis *f. sp. *poae *on bluegrass (*Poa pratensis*) and *P. striiformis *f. sp. *dactylidis *on orchard grass (*Dactylis glomerata*) [9,32]. Among the various formae speciales, the wheat and barley stripe rust pathogens are most economically important. Wheat stripe rust has been reported in more than 60 countries and all continents except Antarctica [6]. Devastating epidemics of wheat stripe rust often occur in many countries in Africa, Asia, Australia, Europe, North America and South America [6,32]. In the U. S., stripe rust of wheat has existed for more than 100 years [19,25]. The disease had been primarily a major problem in western US before 2000, but has become increasingly important in the south central and the Great Plains since 2000 [6,11,25]. Barley stripe rust is a relatively new disease in the west hemisphere. It has caused severe damage in some locations since it was introduced to Colombia in 1975 from Europe [14], and spread to Mexico in 1987 [1] and the U. S. in 1991 [5,9,29]. In spite of its importance, very little is known about the molecular biology and the genomics of the stripe rust fungus.

The life cycle of the stripe rust fungus consists of the dikaryotic uredial and diploid telial stages in the nature [24,32]. Teliospores can germinate to form haploid basidiniospores. Unlike the stem rust (*P. graminis*) and leaf rust (*P. triticina*) pathogens, the stripe rust pathogen does not have known alternate hosts for basidiniospores to infect, and thus, it does not have known sexual pycnial and aecial stages. Therefore, isolates of the fungus cannot be crossed through sexual hybridization, which makes it impossible to study the fungal genes through classic genetic approaches. The fungus reproduces and spreads through urediniospores and survives as mycelium in living host plants. Because urediniospores cannot keep their viability for very long, living plants (volunteers of wheat and barley crops and grasses, or crops and grasses in cool regions in the summer and in warm regions in the winter) are essential to keep the fungus alive from season to season. Although the pathogen does not have known sexual reproduction, there is a high degree of variation in virulence and DNA polymorphism in the natural populations of the stripe rust pathogens [5,6,8,9,11,25]. More than 100 races of *P. striiformis *f. sp. *tritici *and more than 70 races of *P. striiformis *f. sp. *hordei *have been identified in the U. S. [5,6] based on virulence/avirulence patterns produced on differential cultivars by isolates of the pathogens. The avirulence or virulence phenotypes have not been associated with any specific genes or DNA sequences due to the factors that the pathogen can not be studied by conventional analyses.

The expressed sequence tag (EST) technology is an approach to identify genes in organisms that are difficult to study using classic genetic approaches and gene mutation by insertional mutagenesis. Liu et al. [26] analyzed abundant and stage-specific mRNA from *P. graminis*. Lin et al. [23] isolated and studied the expression of a host response gene family encoding thaumatin-like proteins in incompatible oat-stem rust fungus interactions. Recently, EST libraries have been constructed for various fungal species including *P. triticina *[18], the probably most closely related fungal species to *P. striiformis*. ESTs provide valuable putative gene sequence information for genomic studies of targeted organisms. However, EST data has its own limitations such as incomplete cDNA sequence. Because ESTs are typically generated from the 3' end sequences of cDNA clones, EST libraries tend to be incomplete at the 5' end of the transcripts. The cDNA libraries constructed by conventional methods [17] normally contain a high percentage of 5' truncated clones due to the premature stop of reverse transcription (RT) of the template mRNA, particularly for cDNA clones derived from large mRNA molecules and those with the potential to form secondary structures. The size bias against large fragments commonly exists in conventional cDNA cloning procedures. Certain limitations also apply to the end products of the automatic EST assemblies, which may be composed of ESTs generated from different tissues or different developmental stages and may not reflect the accurate transcripts.

Several methods have been developed to construct cDNA libraries that are enriched for full-length cDNAs, including RNA oligo ligation to the 5' end of mRNA [21,33], 5' cap affinity selection via eukaryotic initiation factor [15], or 5' cap biotinylation followed by biotin affinity selection [2]. These methods can be used to improve the full-length cDNA clone content of the cDNA library, but they are all very laborious and involve several enzymatic steps that must be performed on mRNA. Therefore, they are prone to quality loss through RNA degradation. Furthermore, they all require high amounts of starting mRNA at μg level for reverse transcription and cloning processes. Comprehensive sets of accurate, full-length cDNA sequences would address many of the current limitations of the EST data. Genome-scale collections of full-length cDNA become important for analyses of the structures and functions of expressed genes and their products [31]. Full-length cDNA library is a powerful tool for functional genomics and is widely used as physical resources for identifying genes [36].

A full-length cDNA library should be an important resource for studying important genes of the *P. striiformis *pathogen, for sequencing the whole genome, and for determining its interaction with host plants. The objectives of the present study were to construct a full-length cDNA library for *P. striiformis *f. sp. *tritici *and characterize selected cDNA sequences in the library to identify putative functional genes of *P. striiformis *f. sp. *tritici*.

## Results

### Full-length cDNA library generation and characterization

Total RNA was extracted from 30 mg urediniospores of race PST-78 of *P. striiformis *f. sp. *tritici *and yielded approximately 7.5 μg total RNA of high purity. Full-length cDNA was synthesized by reverse transcription and enriched by subsequent long distance PCR (LD PCR). Only non-truncated first strand cDNAs were tagged by the SMART IV oligonucleotide sequence : 5'-AAGCAGTGGTATCAACGCAGAGTGGCCATTACGGCCGGG-3' during the initial reverse transcription. The PCR amplification products were digested with restriction enzyme *sfi*I to generate directional cloning ends. The agarose gel analysis of the digestion showed a significant amount of double stranded cDNA that appeared as a smear ranging from 300 bp to 12 kb. The *sfi*I-digested double strand cDNA was obtained from 5 fractionated gel zones. The gel zones containing smaller cDNA fragments (ranging from 500 bp to 4 kb) yielded approximately 800 ng to 1 μg of cDNA while the gel zones containing large cDNA fragments (ranging from 5 kb to 10 kb) had relatively lower cDNA yields in the 50 – 100 ng range. Although the large cDNA fragment output was relatively low, it was adequate for the subsequent ligation reaction for cloning.

Fractionated cDNA was cloned into the *sfi*I sites of the pDNR-LIB cloning vector and transformed into DH10B competent cells. One microliter of ligation yielded a range of 1,000 to 2,000 recombinant clones for cDNA inserts within the large fractionated gel zone. More than 3,000 recombinant clones were obtained for cDNA inserts from the medium and smaller fractionated gel zones. The clone evaluation of random samples revealed cDNA insert length ranging from 200 bp up to 9 kb across all the fractionation inserts. In general, most of the inserts were in the length range of 500 bp to 4 kb. Large scale transformation was conducted using ligation reactions from each of the fractions, and clones were picked in a mixed fashion using an automated robotic clone picker. A total of 42,240 cDNA clones were arrayed in 112 micro-plates of 384-wells each. An additional copy of the cDNA library was generated by manual duplication.

**Figure 1:**
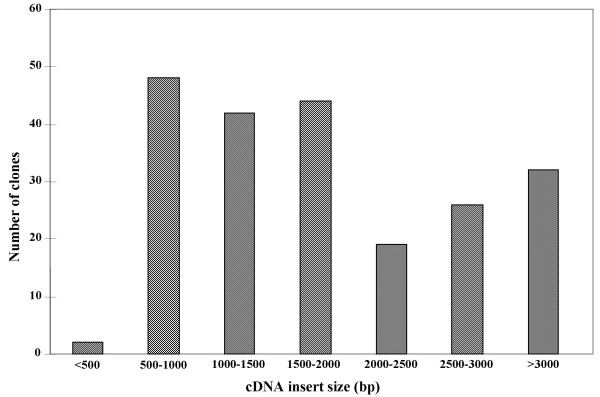
**The insert size distribution of urediniospore cDNA clones of *Puccinia striiformis *f. sp. *tritici***. The insert sizes of 320 randomly picked cDNA clones were determined by *Hin*dIII/*Eco*RI double digestion.

The average cDNA insert size and their distribution were analyzed by random sampling of cDNA clones from randomly selected plates. A total of 320 cDNA clones were double-digested by *Hin*dIII/*Eco*RI. The average cDNA insert size was 1.9 kb. Approximately, 96% of the clones had inserts longer than 500 bp, 54% of the cDNA clones had inserts longer than 1.5 kb, and 15% of the clones contained inserts longer than 3 kb. Only 3% of the clones had inserts smaller than 500 bp (Fig. 1). Therefore, the size fractionation procedure used in this library construction was effective for obtaining cDNA inserts of different lengths.

### cDNA sequence analysis

A total of 198 cDNA clones were sequenced with a single pass reading from both ends of the cloning sites. Sequence reads of 800 – 1,000 bp were achieved for most of the clones. For each sampled cDNA clone, two sequence reads from both ends were aligned and were comparatively edited to generate a consensus sequence contig. Of the 196 clones, we obtained a completed cDNA sequence for 149 clones. The remaining 47 cDNA clones had two partial sequences because they had insert sizes that exceeded the single pass sequencing capability. The 243 single sequences were deposited in the EST sequence database of the GenBank (Accession numbers EG374272 – EG374514).

All edited sequence contigs were searched against the NCBI fungal gene databases and the all-organism gene databases with their translated amino acid sequences. We consider that if a cDNA clone of *P. striiformis *f. sp. *trtici *and a gene in the fungal database share homology significant at an e-value of <1.00E-5, they likely belong to the same gene family and should share a similar broad sense function. A total of 73 cDNA clones (36.9%) met this requirement, and therefore, were considered with functions identified, of which 50 clones had completed sequences, 13 clones had partial sequences that hit the same or similar genes, and 10 clones had one partial sequence hitting a characterized gene (Table 1). These genes represented 51 different protein products that are involved in amino acid metabolism, cell defense, cell cycle, cell signaling, cell structure and growth, energy cycle, lipid and nucleotide metabolism, protein modification, ribosomal protein complex, sugar metabolism, transcription factor, transport metabolism and virulence/infection. Examples of these genes are glycine hydroxymethyltransferase, saccharopine dehydropine, mitogen-activated protein kinase (MAPK), serine/threonine kinase, β-tubulin, deacetylase, mitochondrial ATPase alpha-subunit, fatty acid oxidoreductase, phosphatidyl synthase, endopeptidase, elongation factor, ribosomal RNA unit, glucose-repressible protein, transaldolase, TATA-box binding protein, cell wall glucanase and pectin lyase. Thirty-seven clones (18.9%) had certain levels of homology to genes in other fungi, but the significance levels were not adequate for considering the functions identified (Table 2). Sequences of 36 clones (18.4%) were homologous to fungal genes with functions unclassified and the most of them were hypothetical proteins. Although many of the hypothetical protein genes had e-value < 1.00E-05, they are listed in Table 2 because of their unclear functions. Some of the hypothetical protein genes were homologous to genes in other plant pathogens, such as *Ustilago maydis*, *Gibberella zeae *and *Magnapothe grisea*. These genes could be related to plant infection. Many of the cDNA clones had homology of various levels to genes from plants (12%), other eukaryotes (34%), or to proteins of bacterial origin (11%) (data not shown). There were 50 clones (25.5%) with full-length sequences resulting in no-hit, indicating that they had no homology to any sequence available in the current NCBI databases (Table 3). These genes could be unique to *P. striiformis *f. sp. *tritici*. Alternatively, similar genes in other fungi have not been identified or desposited into the databases.

**Table 1:** Putative genes idenitified in cDNA clones of *Puccinia striiformis *f. sp. *tritici *based on their sequence comparison with other fungal genes through Blastx search of the NCBI databases

Category & clone no.	GenBank accession	Size (bp)	Full length or partial^a^	Best hit in the NCBI fungal databases			
				
				Protein	Accession	Organism	e-value
*1. Amino acid metabolism*							
65N4	EG374380	2044	F	Glycine hydroxymethyltransferase	gb|AAW45780.1	*Cryptococcus neoformans*	1.00E-156
60J18a	EG374421	1142	P	Potential kynurenine 3-monooxygenase	gb|EAK98864.1	*Candida albicans*	2.00E-06
60J18b	EG374422	1220	P	Potential kynurenine 3-monooxygenase	gb|EAK98864.1	*Candida albicans*	1.00E-12
58D15a	EG374299	897	P	Saccharopine dehydrogenase	gi|70993695	*Aspergillus fumigatus*	2.00E-55
58D15b	EG374300	780	P	Spermidine synthase	emb|CAD71251.1	*Neurospora crassa*	3.00E-78
*2. Cell Defense*							
35A16	EG374447	1351	F	Related to stress response protein	emb|CAD21425.1	*Neurospora crassa*	2.00E-23
*3. Cell division/cycle*							
80F12	EG374389	1560	F	Cell division control protein	gb|AAB69764.1	*Candida albicans*	2.00E-28
65O23	EG374383	2037	F	Cyclin c homolog 1	ref|NP_596149.1	*Schizosaccharomyces pombe*	3.00E-07
*4. Cell signaling/cell communication*							
40D3	EG374466	1534	F	Autophagy-related protein	gb|AAW43831.1	*Cryptococcus neoformans*	6.00E-45
70C17a	EG374441	1206	P	Fasciclin I family protein	gi|44890027	*Aspergillus fumigatus*	3.00E-06
58J15b	EG374311	807	P	GTPase activating protein	gb|AAW43777.1	*Cryptococcus neoformans*	2.00E-09
55B10a	EG374277	861	P	MAP kinase 1	gb|AAO61669.1	*Cryptococcus neoformans*	3.00E-19
55B10b	EG374278	932	P	MAP kinase	gb|AAU11317.1	*Alternaria brassicicola*	7.00E-74
65M20	EG374379	1098	F	Nucleoside-diphosphate kinase	emb|CAD37041.1	*Neurospora crassa*	9.00E-53
70E5	EG374404	1766	F	Serine/threonine kinase	gi|58262703	*Cryptococcus neoformans*	3.00E-61
10D13a	EG374414	1122	P	Serine palmitoyl transferase subunit	gb|AAP47107.1	*Aspergillus nidulans*	4.00E-27
10D13b	EG374416	1170	P	Serine palmitoyl transferase subunit	gb|AAP47107.1	*Aspergillus nidulans*	2.00E-18
30G12	EG374337	1131	F	Signal peptidase 18 KD subunit	emb|CAE76335.1	*Neurospora crassa*	3.00E-10
*5. Cell Structure and growth*							
58H22a	EG374306	920	P	Beta-tubulin	emb|CAC83953.1	*Uromyces viciae-fabae*	3.00E-72
58H22b	EG374307	859	P	Beta-tubulin	emb|CAC83953.1	*Uromyces viciae-fabae*	5.00E-68
10I12	EG374325	1105	F	Conidiation protein 6	emb|CAD70456.1	*Neurospora crassa*	2.00E-10
30J9	EG374343	1302	F	Deacetylase	emb|CAD10036.1	*Cryptococcus neoformans*	2.00E-43
60C15	EG374348	1456	F	Deacetylase	gb|AAW47023.1	*Cryptococcus neoformans*	6.00E-35
65D17	EG374372	1449	F	Deacetylase	emb|CAD10036.1	*Cryptococcus neoformans*	4.00E-36
40F18	EG374469	1117	F	Deacetylase	emb|CAD10036.1	*Cryptococcus neoformans*	2.00E-31
55D17	EG374475	1619	F	Deacetylase	emb|CAD10036.1	*Cryptococcus neoformans*	5.00E-18
35C19b	EG374494	836	P	Deacetylase	emb|CAD10036.1	*Cryptococcus neoformans*	6.00E-18
10C3	EG374321	1479	F	Deacetylase	gb|AAW47023.1	*Cryptococcus neoformans*	6.00E-26
35N24	EG374461	783	F	Hydrophobin	emb|CAD42710.1	*Davidiella tassiana*	5.00E-34
32H21a	EG374436	1176	P	Intraorganellar peroxisomal translocation component Pay32p (PAY32) gene	gi|5821763	*Yarrowia lipolytica*	4.00E-32
40B22	EG374465	1708	F	Nuclear filament-containing protein	emb|CAA93293.1|	*Schizosaccharomyces pombe*	5.00E-16
35G11a	EG374497	819	P	Pria_lened pria protein	emb|CAA43289.1	*Lentinula edodes*	2.00E-12
65M2	EG374413	2097	F	UDP-glucose dehydrogenase	gb|AAS20528.1	*Cryptococcus neoformans*	1.00E-145
*6. Energy/TCA cycle*							
35D23b	EG374496	629	P	64 kDa mitochondrial NADH dehydrogenase	gb|AAW44492.1	*Cryptococcus neoformans*	1.00E-07
40H12	EG374471	1249	F	Iron-sulfur cluster Isu1-like protein	gb|AAQ98966.1	*Cryptococcus neoformans*	8.00E-56
55E23a	EG374279	957	P	Mitochondrial ATPase alpha-subunit	gb|AAA33560.1	*Neurospora crassa*	6.00E-78
55E23b	EG374280	870	P	Mitochondrial ATPase alpha-subunit	gb|AAA33560.1	*Neurospora crassa*	1.00E-101
90M15	EG374409	1570	F	Mitochondrial carrier family protein	gb|EAK95613.1	*Candida albicans*	1.00E-46
30N15a	EG374419	1078	P	Succinate dehydrogenase flavoprotein subunit precursor	gb|AAW45324.1	*Cryptococcus neoformans*	1.00E-63
30N15b	EG374420	1143	P	Succinate dehydrogenase flavoprotein subunit precursor	gb|AAW45324.1	*Cryptococcus neoformans*	1.00E-136
10A2	EG374481	1114	F	V-type ATPase subunit G	gb|AAB41886.1|	*Neurospora crassa*	6.00E-15
*7. Lipid metabolism*							
65D3	EG374370	1809	F	Diacylglycerol O-acyltransferase	gi|58268157	*Cryptococcus neoformans*	1.00E-84
65G21a	EG374424	1078	P	Fatty acid oxidoreductase	gb|AAW46114.1	*Cryptococcus neoformans*	2.00E-05
65G21b	EG374425	1149	P	Fatty acid oxidoreductase	gb|AAW46114.1	*Cryptococcus neoformans*	3.00E-32
58J11b	EG374309	732	P	Phosphatidyl synthase	gi|70999337	*Aspergillus fumigatus*	2.00E-20
*8. Nucleotide metabolism*							
58C19a	EG374297	827	P	Uracil DNA N-glycosylase	gb|AAW41098.1	*Cryptococcus neoformans*	7.00E-16
58C19b	EG374298	857	P	Uracil DNA N-glycosylase	gb|AAW41098.1	*Cryptococcus neoformans*	1.00E-19
*9. Protein modification*							
65B1	EG374366	1847	F	Carboxypeptidase	gi|19115337	*Schizosaccharomyces pombe*	7.00E-06
66B11a	EG374437	1145	P	Endopeptidase	gb|AAW41068.1	*Cryptococcus neoformans*	2.00E-69
66B11b	EG374438	1200	P	Endopeptidase	gb|AAW41068.1	*Cryptococcus neoformans*	1.00E-48
80N15	EG374397	1944	F	Translation elongation factor eEF-1 alpha chain	pir||S57200	*Puccinia graminis*	0.00E+00
*10. Protein translational modification*							
55N13	EG374483	833	F	Ubiquitin-conjugating enzyme	ref|NP_594859.1	*Schizosaccharomyces pombe*	7.00E-21
*11. Ribosomal protein complex*							
55B4	EG374472	770	F	16S small subunit ribosomal RNA	gi|52699765	*Xanthoria elegans*	2.00E-08
35O22	EG374462	938	F	18S ribosomal RNA	gi|21702995	*Gymnosporangium libocedri*	1.00E-154
60E22	EG374352	1117	F	18S ribosomal RNA	gi|34493860	*Puccinia graminis *f. sp.*tritici*	3.00E-142
65C12	EG374368	1136	F	18S ribosomal RNA	gi|34493860	*Puccinia graminis *f. sp.*tritici*	2.00E-66
90D5a	EG374432	1119	P	18S ribosomal RNA	gi|21724233	*Puccinia striiformis *f. sp.*tritici*	6.00E-102
90D5b	EG374431	1147	P	ITS1, ITS2 and 5.8S ribosomal RNA	gi|3668067	*Tricholoma matsutake*	9.00E-54
58E11b	EG374302	831	P	25S ribosomal RNA	gi|169606	*Puccinia graminis f. sp. dactylis*	1.00E-09
23H10b	EG374283	1921	F	28S ribosomal RNA	gi|37703614	*Puccinia allii*	1.00E-83
35M12a	EG374458	763	F	28S ribosomal RNA	gi|21724230	*Puccinia graminis *f. sp. *tritici*	2.00E-14
35N2	EG374460	917	F	28S ribosomal RNA	gi|46810582	*Fuscoporia viticola*	4.00E-06
35P13	EG374463	888	F	28S ribosomal RNA	gi|86160913	*Melampsora epitea*	2.00E-16
40A4	EG374464	951	F	28S ribosomal RNA	gi|58532805	*Puccinia carthami*	4.00E-05
55J11	EG374479	957	F	28S ribosomal RNA	gi|21724233	*Puccinia striiformis f. sp. tritici*	2.00E-26
35I10b	EG374502	422	P	28S ribosomal RNA	gi|21914221	*Puccinia graminis*	5.00E-77
35I22a	EG374505	716	P	28S ribosomal RNA	gi|21914221	*Puccinia graminis*	2.00E-70
35I22b	EG374504	878	P	ITS1, ITS2 and 5.8S ribosomal RNA	gi|21724233	*Puccinia striiformis *f. sp.*tritici*	5.00E-134
10G18	EG374323	1108	F	28S ribosomal RNA	gi|84452427	*Cladosporium cladosporioides*	1.00E-59
30C19	EG374333	1117	F	28S ribosomal RNA	gi|62005831	*Puccinia ferruginosa*	2.00E-13
30H3	EG374340	1052	F	28S ribosomal RNA	gi|21724233	*Puccinia striiformis f. sp. tritici*	3.00E-71
30I12	EG374341	1067	F	28S ribosomal RNA	gi|21724233	*Puccinia striiformis f. sp. tritici*	2.00E-39
30M20	EG374347	1008	F	28S ribosomal RNA	gi|21914221	*Puccinia graminis*	1.00E-93
60J23	EG374357	2112	F	calnexin	gb|AAS68033.1	*Aspergillus fumigatus*	1.00E-133
*12. Sugar/glycolysis metabolism*							
30I15b	EG374418	617	P	Glucose-repressible protein	emb|CAC28672.1	*Neurospora crassa*	2.00E-14
90C20	EG374401	1130	F	Glucose-repressible protein	gi|70996962	*Aspergillus fumigatus*	7.00E-18
55J22b	EG374287	887	P	Glyoxal oxidase precursor	gb|AAW44259.1	*Cryptococcus neoformans*	2.00E-90
55J22a	EG374286	764	P	Glyoxal oxidase precursor	gb|AAW41343.1	*Cryptococcus neoformans*	3.00E-30
90H16	EG374405	1753	F	Phosphopyruvate hydratase	gi|1086120	*Cladosporium herbarum*	1.00E-139
30K8	EG374344	1547	F	Transaldolase	gb|AAW46393.1	*Cryptococcus neoformans*	3.00E-95
*13. Transcription factor*							
58E6	EG374485	1310	F	TATA-box binding protein	gb|AAB57876.1	*Emericella nidulans*	7.00E-63
*14. Transport metabolism*							
65M6	EG374378	1119	F	Cation transport-related protein	gb|AAW42114.1	*Cryptococcus neoformans*	3.00E-13
15. virulence/infection related protein							
70I2	EG374433	1952	F	Cell wall glucanase	gi|70998053	*Aspergillus fumigatus*	2.00E-25
30M9	EG374345	1162	F	Differentiation-related/infection protein	gb|AAD38996.1	*Uromyces appendiculatus*	7.00E-11
80C7	EG374385	1180	F	Differentiation-related/infection protein	gb|AAD38996.1	*Uromyces appendiculatus*	1.00E-10
60E18	EG374351	2147	F	Pectin lyase	gb|AAA21817.1	*Glomerella cingulata*	2.00E-06

^a ^F = full-length sequence and P = partial sequence.

**Table 2:** cDNA clones showing homology to genes with characterized or unclassified proteins through Blastx search of the NCBI fungal databases

Category & clone no.	GenBank accession	Size (bp)	Full length or partial^a^	Best hit in the NCBI databases			
				Protein	Accession	Organism	e-value
*1. Amino acid metabolism*							
35I14	EG374455	766	F	Cystathionine beta-lyase	gi|6636350	*Botryotinia fuckeliana*	5.70E+00
*2. Cell Defense*							
66C24a	EG374440	1175	P	88 kDa immunoreactive mannoprotein MP88	gb|AAL87197.1	*Cryptococcus neoformans*	1.00E-03
*3. Cell Division/cycle*							
10F19	EG374412	1877	F	g1/s-specific cyclin pcl1 (cyclin hcs26)	gb|AAW44590.1	*Cryptococcus neoformans*	2.00E-04
*4. Cell signaling/cell communication*							
65G15	EG374514	1106	P	Protein kinase	gi|15072451	*Cryphonectria parasitica*	1.20E+00
30E21	EG374336	1128	F	Serine/threonine kinase	gi|22531808	*Ustilago maydis*	3.90E-01
65C6	EG374367	1649	F	Serine/threonine phosphatase	gi|33087517	*Hypocrea jecorina*	3.90E-01
80G5b	EG374428	1230	P	Mitogen-activated protein kinase	gi|57227328	*Cryptococcus neoformans*	1.70E-00
*5. Cell Structure and growth*							
58G9	EG374486	1714	F	Beta tubulin	gi|47834278	*Penicillium flavigenum*	6.40E-00
40G6b	EG374274	888	P	Cell wall protein	gi|68471254	*Candida albicans*	4.60E-01
58C4b	EG374296	819	P	Cell surface protein	gi|70983232	*Aspergillus fumigatus*	2.60E-02
10D19	EG374322	1212	F	Cell wall mannoprotein	ref|NP_012685.1	*Saccharomyces cerevisiae*	1.00E-03
90I19	EG374406	1240	F	Cell wall mannoprotein	gi|6322611	*Saccharomyces cerevisiae*	1.50E-02
90C22	EG374402	1641	F	Cytoplasm protein	gb|AAW42379.1	*Cryptococcus neoformans*	1.00E-04
10I15	EG374326	1088	F	Mitochondrial outer membrane beta-barrel protein	gi|45758780	*Neurospora crassa*	1.70E-01
60H1	EG374354	1035	F	Nuclear pore complex subunit	gi|46437749	*Candida albicans*	5.00E-00
70I19a	EG374443	1132	P	Nucleoskeletal-like protein	gi|172053	*Saccharomyces cerevisiae*	1.30E-01
*6. Differentiation- related protein*							
70A18	EG374371	1207	F	Differentiation-related protein	gb|AAD38996.1	*Uromyces appendiculatus*	6.00E-03
*7. Mating type*							
30M10	EG374346	1025	F	Mating type alpha locus	gi|73914085	*Cryptococcus gattii*	6.80E+00
30C22	EG374334	1110	F	Mating type alpha locus	gi|73914085	*Cryptococcus gattii*	7.50E+00
*8. Nucleotide metabolism*							
35K8	EG374456	1572	F	Ribonuclease H2 subunit	gi|6320485	*Saccharomyces cerevisiae*	9.00E+00
*9. Protein translational modification*							
100C10	EG374490	1179	F	Non-ribosomal peptide synthetase	gi|62006079	*Hypocrea virens*	1.20E+00
*10. Ribosomal protein complex*							
35L17	EG374457	585	F	18S ribosomal RNA	gi|51102377	*Microbotryum dianthorum*	4.20E-02
40C19a	EG374512	706	P	18S ribosomal RNA	gi|28412377	*Leotiomycete sp*.	5.40E-01
35H2b	EG374500	786	P	26S large subunit ribosomal RNA	gi|30313824	*Pichia guilliermondii AjvM13*	1.00E-03
35E4	EG374451	897	F	28S ribosomal RNA	gi|46810582	*Fuscoporia viticola*	5.00E-03
35P11a	EG374506	667	P	28S ribosomal RNA	gi|62005826	*Puccinia artemisiae-keiskeanae*	1.00E-04
55B15	EG374473	954	F	28S ribosomal RNA	gi|84794517	*Puccinia striiformoides*	3.60E-01
58B3	EG374484	884	F	28S ribosomal RNA	gi|46810582	*Fuscoporia viticola*	3.30E-01
58N22	EG374488	996	F	28S ribosomal RNA	gi|20452324	*Rhodotorula pilati*	3.30E-01
66I12	EG374338	1167	F	28S ribosomal RNA	gi|46810582	*Fuscoporia viticola*	3.00E-04
80G5a	EG374427	1106	P	Calnexin	gi|45551624	*Aspergillus fumigatus*	2.30E-00
*11. Sugar/glycolysis metabolism*							
58G18b	EG374304	796	P	Pyruvate decarboxylase	gi|68480982	*Candida albicans*	1.40E+00
10N6	EG374330	1029	F	Pyruvate kinase	gi|168073	*Aspergillus nidulans*	6.00E+00
*12. Transport metabolism*							
30G15	EG374339	1087	F	Membrane zinc transporter	gi|47156070	*Aspergillus fumigatus*	5.70E-01
40H8a	EG374275	656	P	amino acid transporter	gi|70985369	*Aspergillus fumigatus*	3.10E+00
80K19	EG374395	1728	F	Na+-ATPase	gi|1777377	*Zygosaccharomyces rouxii*	2.00E-04
55L18b	EG374289	845	P	Peptide transporter	gi|70982509	*Aspergillus fumigatus*	5.30E-01
*13. Unclassified*							
80G10	EG374391	1132	F	Genomic sequence	gi|48056381	*Phakopsora pachyrhizi*	7.00E-53
04F9	EG374470	1127	F	Hypothetical protein	gi|71006713	*Ustilago maydis*	1.00E-06
10N10	EG374331	1106	F	Hypothetical protein	gi|58258450	*Cryptococcus neoformans*	6.00E-22
30I21	EG374342	1906	F	Hypothetical protein	gi|71023234	*Ustilago maydis*	1.00E-21
35B6	EG374449	1060	F	Hypothetical protein	gb|EAA67250.1	*Gibberella zeae*	1.00E-03
35C10	EG374450	1465	F	Hypothetical protein	gi|71004383	*Ustilago maydis 521*	2.00E-08
35G21	EG374454	1332	F	Hypothetical protein	gb|EAK81105.1	*Ustilago maydis*	5.00E-09
35H2a	EG374499	758	P	Hypothetical protein	gi|71021872	*Ustilago maydis*	1.80E+00
40B2a	EG374508	603	P	Hypothetical protein	gi|85114517	*Neurospora crassa*	3.00E-05
40C12a	EG374510	792	P	Hypothetical protein	gi|71019552	*Ustilago maydis*	4.00E-01
55L8	EG374491	1417	F	Hypothetical protein	gi|71004813	*Ustilago maydis*	1.50E-01
58C4a	EG374296	764	P	Hypothetical protein	MGG_09875.5^b^	*Magnaporthe grisea*	6.00E-12
60D4	EG374350	1123	F	Hypothetical protein	gi|50259357	*Cryptococcus neoformans*	7.00E-04
60I14	EG374356	1565	F	Hypothetical protein	gi|58263159	*Cryptococcus neoformans*	2.00E-09
60L15	EG374359	2073	F	Hypothetical protein	gb|EAA47832.1	*Magnaporthe grisea*	7.00E-10
60N2	EG374363	1109	F	Hypothetical protein	gi|46096746	*Ustilago maydis*	7.00E-03
60N6	EG374364	1071	F	Hypothetical protein	gi|49642978	*Kluyveromyces lactis*	8.00E-17
65H5	EG374374	1390	F	Hypothetical protein	gi|85095053	*Neurospora crassa*	1.40E+00
65I3	EG374375	1870	F	Hypothetical protein	gb|EAK86140.1	*Ustilago maydis*	1.00E-129
65O15	EG374381	1893	F	Hypothetical protein	gi|71006255	*Ustilago maydis*	1.10E+00
66B6	EG374316	1263	F	Hypothetical protein	gb|EAK81690.1	*Ustilago maydis*	1.00E-03
66B11a	EG374437	1145	P	Hypothetical protein	AN2903.3^b^	*Aspergillus nidulans*	3.00E-57
66B11b	EG374438	1200	P	Hypothetical protein	FG10782.1^b^	*Fusarium graminearum*	5.00E-49
66C18	EG374327	2043	F	Hypothetical protein	gb|EAA59593.1	*Aspergillus nidulans*	2.00E-12
70A3	EG374360	1835	F	Hypothetical protein	SS1G_14513.1^b^	*Sclerotinia sclerotiorum*	8.00E-18
70C17b	EG374442	1191	P	Hypothetical protein	AN0768.3^b^	*Aspergillus nidulans*	1.00E-07
70H16	EG374426	1121	F	Hypothetical protein	gi|38100779	*Magnaporthe grisea*	2.60E+00
70I19b	EG374443	1190	P	Hypothetical protein	NCU02808.2^b^	*Neurospora crassa*	2.00E-08
70K15b	EG374320	933	P	Hypothetical protein	gi|58261561	*Cryptococcus neoformans*	1.00E-07
70L24b	EG374446	1168	P	Hypothetical protein	gb|EAA28928.1	*Neurospora crassa*	3.00E-23
80I9	EG374394	1060	F	Hypothetical protein	gi|58259618	*Cryptococcus neoformans*	1.50E+00
90O3	EG374410	1725	F	Hypothetical protein	gi|85119288	*Neurospora crassa*	1.20E-02
90O18	EG374411	1973	F	Hypothetical protein	CHG04543.1^b^	*Chaetomium globosum*	4.00E-07
66C24b	EG374440	1271	P	Macrofage activating glycoprotein	gi|15722495	*Cryptococcus neoformans*	3.00E-08
30E3	EG374335	1406	F	Probable gEgh 16 protein	emb|CAE85538.1	*Neurospora crassa*	8.00E-07
60I8	EG374355	1039	F	Related to ars binding protein 2	gi|18376044	*Neurospora crassa*	6.60E+00
55J15b	EG374285	896	P	Telomeric sequence DNA	gi|173051	*Saccharomyces cerevisiae*	2.00E-05
55E7	EG374477	1253	F	Unknown protein in chromosome E	gi|49654999	*Debaryomyces hansenii*	3.00E-06
55F15a	EG374281	461	P	Unknown protein in chromosome G	gi|50427978	*Debaryomyces hansenii*	2.00E-03
60L20	EG374361	1646	F	Unknown protein in chromosome VI	gi|39975020	*Magnaporthe grisea*	3.00E-18
60N1	EG374362	2024	F	Unknown protein in chromosome 1	gi|46110618	*Gibberella zeae*	2.00E-09
70F20	EG374415	1818	F	Unknown protein in chromosome III	gi|58270250	*Magnaporthe grisea*	1.60E+00
80M4	EG374396	1985	F	Unknown protein in chromosome G	gi|49657202	*Debaryomyces hansenii*	1.00E-03
80N10	EG374430	563	P	Phytochrome	gi|57337632	*Emericella nidulans*	4.30E-00
90B8	EG374400	2011	F	Unknown protein in chromosome G	gi|49657202	*Debaryomyces hansenii*	4.90E-02
90L21	EG374408	2002	F	Unknown protein in chromosome A	gi|49524079	*Candida glabrata*	1.20E+00

^a ^F = full-length sequence and P = partial sequence.

^b ^Data generated from Blastx search of the fungal database of the Broad Institute [34].

**Table 3:** cDNA clones that produced no hit in the Blastx search of the NCBI fungal databases

Category & Clone no.	GenBank accession	Size (bp)	Full length or partial^a^	Category & clone no.	GenBank accession	Size (bp)	Full length or partial^a^
04A1	EG374448	1188	F	55N9	EG374482	1171	F
04C13	EG374459	1423	F	55B9a	EG374292	585	P
04P11	EG374434	1133	F	55B9b	EG374293	930	P
100B17	EG374489	1137	F	58E11a	EG374301	542	P
10B5	EG374492	1161	F	58G18a	EG374303	791	P
10C11	EG374503	1235	F	58J11a	EG374308	672	P
10I7	EG374324	1112	F	58J15a	EG374310	921	P
10K3	EG374328	1687	F	58L3	EG374487	959	F
10L3	EG374272	1099	F	58M15a	EG374314	719	P
10N5	EG374329	1090	F	58M15b	EG374315	718	P
10O19	EG374332	1359	F	58M7a	EG374312	788	P
30I15a	EG374417	1032	P	58M7b	EG374313	934	P
32B15	EG374294	1296	F	58N10a	EG374317	287	P
32H21b	EG374436	1249	P	58N10b	EG374318	837	P
35C19a	EG374493	739	P	60F10	EG374353	1131	F
35D23a	EG374495	775	P	60L12	EG374358	1239	F
35F14	EG374453	971	F	60O23	EG374365	1084	F
35F7	EG374452	1086	F	65C23	EG374369	2047	F
35G11b	EG374498	757	P	65G1	EG374373	1631	F
35I10a	EG374501	807	P	65G15b	EG374514	1158	P
35P11b	EG374507	682	P	65I10	EG374376	1010	F
40B2b	EG374509	860	P	65K18	EG374377	1230	F
40C12b	EG374511	921	P	65P1	EG374384	1814	F
40C19b	EG374513	857	P	66M21	EG374349	1437	F
40E10	EG374467	713	F	70C4	EG374382	1518	F
40E23	EG374468	734	F	70D12	EG374393	1285	F
40G6a	EG374273	779	P	70K15a	EG374319	722	P
40H8b	EG374276	811	P	70L24a	EG374445	1104	P
50M2	EG374305	1182	F	80D10	EG374386	1147	F
55C20	EG374474	868	F	80E22	EG374388	2064	F
55E2	EG374476	1272	F	80E4	EG374387	1173	F
55F12	EG374478	935	F	80F15	EG374390	2129	F
55F15b	EG374282	865	P	80G19	EG374392	1124	F
55J15a	EG374284	660	P	80N10a	EG374429	1091	P
55L18a	EG374288	930	P	80O12	EG374398	1517	F
55M5	EG374480	942	F	80O24	EG374399	2098	F
55N22a	EG374290	813	P	90H10	EG374403	1748	F
55N22a	EG374291	282	P	90K17	EG374407	1896	F

^a ^F = full-length sequence and P = partial sequence.

### Identification of open reading frames

Various lengths of open reading frames (ORFs) were identified from 167 cDNA clones using the Lasergene sequence analysis software (DNASTAR package, WI. USA). The quality of the cDNA libraries with respect to the full-length (intactness) of cDNA was evaluated using three parameters: 1) identification of the 5'-end sequence structures of the insert, 2) ATG start site at their 5'-end for complete ORF contents and 3) Blastx evaluation of pre-determined ORF with corresponding amino acid sequences in the GenBank. Multiple ORFs with different length were frequently identified in a given cDNA sequence. When methionine was found aligned (including gaps) with first amino acid of a completed sequence (within the longest ORFs) with the first ATG start codon at the 5' end, a cDNA sequence was determined as a full-length transcript. Most of the cDNA sequences retained the specific 5'-end priming sequences (5'-CGGCCGGG-3'). A total of 128 complete ORFs were identified with first translation initiation codon ATG. The longest ORF was 951 bp, and the shortest ORF was 93 bp. The longest ORF sequence was selected from each analyzed cDNA and validated with the corresponding amino acid sequences to determine the genuine ORF. Four cDNA sequences were identified which contain incomplete ORF sequences, indicating incomplete transcripts for those cDNA clones. Nearly 86% of the cDNA sequences were found containing completed ORFs with a translation initiation codon (ATG). Each of the validated ORFs was able to translate into a continuous protein sequence with a translation initiation codon. This finding indicated high percentage of cDNA clones containing full-length transcripts with various sizes of ORFs in the cDNA library.

## Discussion

A cDNA library can provide molecular resources for analysis of genes involved in the biology of a plant pathogenic fungus, such as genes responsible for the development, survival, pathogenicity and virulence. In order to initiate studies on the basic genome structure and gene expression of *P. striiformis *with infective state, we constructed a full-length cDNA library and a BAC library from urediniospores of a predominant race of *P. striiformis *f. sp. *tritici *[10]. The full-length cDNA library can be used to study the normal transcription profiles for the uredinial state, the biologically and epidemiologically essential stage of the fungus. The current cDNA library will serve as a major genetic resource for identifying and isolating full-length genes and functional units from the *P. striiformis *genome. Because this cDNA library was constructed from urediniospores of the pathogen, it should include expressed genes unique to this spore stage. Therefore, the cDNA library should have avoided EST limitations that are commonly generated by automatic assemblies of transcripts from different tissues. Controlled greenhouse conditions and careful handling of the plants and spores minimized possibility of contaminations by other fungal spores. Powdery mildew or leaf rust, which sometimes contaminates stripe rust spores, were not observed on the stripe rust – sporulating plants. Therefore, genes or cDNA sequences identified in this study should be from urediniospores of *P. striiformis *f. sp. *tritici*. This also was confirmed in a separate study, in which primers of all 12 randomly picked cDNA clones were successfully amplified clones in the BAC library constructed with the same race of the pathogen (data not shown).

A urediniospore of *P. striiformis *is an infectious structure that is critical for the rust to initiate the infection process. Although the fungus produces other spores, teliospores and basidiospores, they do not result in infection of host plants because the fungus does not have alternate hosts for basidiospores to infect. Compared to mycelium, a urediniospore is relatively more resistant to adverse environmental conditions. Therefore, the urediniospore stage should contain most of the pathogen genes involved in the pathogen development, survival and pathogenicity. Thus, our first full-length cDNA library for *P. striiformis *was constructed using urediniospores. Such transcript (gene) collection should include the genes that are important for the unique physical properties and characters of the urediniospores of *P. striiformis*. These genes are essential to maintain their germination and infective abilities. Therefore, the current full-length cDNA library would be one of the useful genomic resources for the functional genomic study of this important agricultural pathogen. Our full-length cDNA library reported here is the first large scale transcript collection for *P. striiformis*. As expression of certain genes are stage-specific and genes involved in plant-pathogen interactions express in haustoria [4,13], currently, we are working together with Scot Hulbert's lab to construct a full-length cDNA library from haustoria of the same stripe rust race used in this study.

The technology used in this study for full-length cDNA enrichment is robust and only requires less than 1 μg of starting total RNA. By using the MMLV reverse transcriptase, only the 5'-end tagged cDNAs are not prematurely terminated and can be amplified into full-length by an RNA oligo-specific primer [35,37]. The size fractionation process was modified in this study to generate large directional full-length cDNA inserts, which enriched full-length cDNA clones to have an insert size up to 9 kb. The enrichment of the full-length cDNA was achieved by PCR amplification following the cDNA synthesis. Because selection bias could favor the smaller cDNA, we used fewer PCR cycles to minimize such bias as previously suggested [35]. The conventionally constructed cDNA libraries rarely carry cDNA inserts over 2 kb, because the longer transcripts are often easily truncated during cDNA synthesis process, causing size bias against the larger cDNA fragments in cloning process. In our study, up to 22 PCR amplification cycles were used to generate adequate amount cDNA for cloning. The evaluation of cDNA insert size and its distribution showed a low level of insert size bias in the final cDNA library. Most of the cDNA inserts ranged from 500 bp to 1,500 bp, and there were high number of cDNA clones harboring inserts over 3,000 bp. Such results indicate that the size fraction is an effective selection approach to ensure the full-length cDNA content level in the cDNA library. The high quality of the initial total RNA and the optimal LD PCR conditions also resulted in low size bias level for the insert size distribution in this library. High quality and adequate amount of the initial mRNA is the key for yielding sufficient amount of the first strand full-length cDNA by reverse transcription. To reduce the redundancy and to avoid underrepresentation of different transcript species, cDNA fragments with different fractionated sizes were balanced and subjected to library construction. A considerable number of clones with an insert over 3 kb were found in our cDNA library, such big insert size is rarely found in conventional cDNA libraries.

The sequences of 5'-end transcripts are important for finding the signals for initiation of transcription. Irrespective of the length of cDNA, identification of the specific 5'-end nucleotide sequences in cDNA is commonly used to determine the full-length cDNA content and quality. In many cases, the 5'-end nucleotide sequences are referred to as a 5' cap structure [3,15,20,27]. We also found that nearly 95% of the cDNA clones contained the known 5'-end sequence : 5'-CGGCCGGG-3' (DB Clontech. USA), where as (G)_3 _at 3'-end will bind to the intact reveres transcripts which has nucleotide priming site CCC at its 5'-end. Completed ORFs were identified in cDNA sequence having the 5'-end sequence structure (5'-CGGCCGGG-3'). Presence of the ATG initiation codon aligned with amino acid methionine also was used as an indicator for the quality of full-length cDNA.

Blastx was used to search the entire NCBI GenBank with e-value of 10^-5^, which revealed 37% of the cDNA clones with high homologies to genes with known functions in the database. The relative low match rate to homologous genes from the blastx search might be due to the lack of gene information in the database for fungi. During the search process, the longest ORFs in each given cDNA sequence was also evaluated with amino acid alignments. The results showed that 86% of the cDNA clones contain ORFs with the translation initiation codon and stop codon. In addition, the existence of multi-exonic structure within some ORFs is additional evidence that supports their biological reality of genes or transcripts. The Kozak rules were found not totally applicable in determining ORFs in this study. Perhaps the Kozak rules are more suitable for analysis of mammalian genomes [22].

So far, there have been no other reports on the genome of *P. striiformis *in relation to function and biology of this important pathogen. In this study, we have identified genes encoding 51 different protein products involved in eleven aspects of the pathogen cell biology and plant infection. These genes are the first group of genes reported for the stripe rust pathogen. The genes identified for virulence/infection can be used in transient expression to confirm their function in pathogenicity. Although we sequenced only a small portion of the cDNA library, the study demonstrated the high efficiency of this procedure for the identification of putative genes of known function. As more and more genes with identified functions from other organisms are deposited into the databases, genes with important functions in *P. striiformis *should be more efficiently identified using our cDNA library. Even though sequences of only 196 clones were characterized in this study, we identified 19 cDNA clones encoding ribosomal RNA subunits, seven clones encoding deacetylase, and two clones encoding the glucose-repressible protein. The results may indicate the mRNA abundance of these genes. In this study, 10 cDNA clones had one of the two partial sequences with high homology (e-value ranging from 3E-06 to 5E-77) to genes identified in other fungi, but another partial sequence produced no hit. The results may indicate that these genes have very long sequences, and also may reflect that similar gene sequences in other fungi are mainly short EST sequences. When blastx search was conducted using other fungal genomic databases [34], seven cDNA clones, which produced no hit when blasted with the NCBI database, were identified to have some homology with unknown functions in various fungal species. In this study, we identified 37.2% of the clones with known genes, 18.4% encoding hypothetical proteins, and 25.5% no hit. These numbers are quite different from the 11%, 23%, and 66% of these categories, respectively, found in the urediniospore EST library of *P. graminis *f. sp. *tritici*, the wheat stem rust pathogen (L. Szabo, personal communication). The differences could be due to the clone sampling sizes of the studies and the different types of libraries (the full-length cDNA library for *P. striiformis *f. sp. *tritici *and conventional EST library for *P. graminis *f. sp. *tritici*). As more genes or ESTs from other *Puccinia *species infecting cereal crops become available, it will be more feasible to identify genes common to this group of the rust pathogens and also identify genes unique to particular species.

## Conclusion

A full-length cDNA library was constructed using urediniospores of the wheat stripe rust pathogen. Using the library, we identified 51 genes involved in amino acid metabolism, cell defense, cell cycle, cell signaling, cell structure and growth, energy cycle, lipid and nucleotide metabolism, protein modification, ribosomal protein complex, sugar metabolism, transcription factor, transport metabolism, and virulence/infection. The results of function-identified genes demonstrated that the full-length library is useful in the study of functional genomics of the important plant pathogenic fungus. Research will be conducted to identify genes involved in the development, survival and pathogenicity of the pathogen using the cDNA library.

## Methods

### Total RNA isolation from urediniospores of *P. striiformis *f. sp. *tritici*

Urediniospores from race PST-78 of *P. striiformis *f. sp. *tritici*, a predominant race of the wheat stripe rust [11], were harvested from infected leaves 15 days after inoculation. The inoculation method and conditions for growing plants before and after inoculation were as described by Chen and Line [7]. For total RNA extraction, approximately 30 mg urediniospores were pre-chilled with liquid nitrogen in a glass vial. Spores were ground in liquid nitrogen with mortar and pestle, and then 10 mM Tris buffer (PH 8.0) was added. Ground frozen powder was transferred to an RNase-free microcentrifuge tube. The SV Total RNA Isolation kit (Pormega. Madison, WI. USA) was used to isolate total RNA from ground urediniospores. The extraction procedure recommended by the kit manufacturer was followed with slight modifications to adapt the use of fungal material. The quantity and purity of isolated total RNA was analyzed by 1% agarose gel electrophoresis and spectrophotometer.

### Full-length cDNA synthesis and size fractionation

First-strand cDNA was synthesized from approximately 500 ng of total RNA using the Creator SMART cDNA Library Construction kit (DB Clontech. USA) following a slightly modified manufacturer's protocol. The first-strand cDNA mixture was used as template to synthesize double-stranded DNA with long distance (LD) PCR. PCR reactions were facilitated by 20 pmol of 5' end PCR primer containing *sfi*I A site (5'AAGCAGTGGTATCAACGCAGAGTGGCCATTACGGCCGGG-3'), and 20 pmol of CDSIII/3' end polyT PCR primers containing *sfi*I B site [5'-ATTCTAGAGGCCGAGGCGGCCGACATG-d(T)_30_N_-1_N-3']. In a 100 μL PCR reaction, 2 μL first-stranded cDNA were used as the template. The PCR reaction mixture contained 20 pmol of 10× PCR buffer, dNTP mix and 5 units of *Taq *polymerase. The LD PCR was performed in a GeneAmp 9600 thermal cycler (ABI Biosystem, USA) with the following program: denature at 95°C for 20 s followed by 22 cycles of 95°C for 5 s, 68°C for 6 min and 4°C soaking. The double stranded cDNA was then treated with proteinase K at 45°C for 20 min to inactivate the remaining DNA polymerase. The double stranded cDNA was then phenol-extracted and precipitated with 10 μL of 3 M sodium acetate, 1.3 μL of glycogen (20 μg/μL) and 2.5 volumes of 100% ethanol. Double stranded cDNA pellet was washed with 80% ethanol, air dried and suspended in 20 μL of water.

Double stranded cDNA was subjected to *sfi*I digestion, 100 μL *sfi*I digestion reaction containing 79 μL of cDNA, 10 μL 10× NE buffer 2 (New England Biolabs, USA) (10 mM Tris-HCl, 50 mM NaCl, 10 mM MgCl_2_, 1 mM dithiothreitol), 1 μL of 100× BSA (100 μg/ml) and 10 units of *sfi*I restriction enzyme (New England Biolabs, USA). Digestion was performed under 50°C for 2 h. Digested cDNA was size-fractionated on 1% agarose gel with 6 V/cm electrophoresis and the size fraction of 500 bp to 10 kb was excised. The excised gel slice was further divided into 5 zones (5 smaller gel slices) corresponding to a cDNA size ranging from 500 bp to 10 kb. Then cDNA in each gel slice was extracted and purified using the MinElute Gel Extraction kit (Qiagen, USA). The final cDNA concentration was adjusted to 5 ng/μl.

### Construction of cDNA library

Approximately 30 ng *sfi*I-digested cDNA fragments were ligated to 100 ng of the pDNR-LIB cloning vector (DB Clontech, USA) using T4 DNA ligase (New England Biolabs, USA) under 16°C for 16 h. The ligation product was directly transformed into competent cell DH10B (Epicentre Technologies, USA) by electroporation. After 1 h SOC recovery incubation, transformed bacterial strain were grown on LB agar plates containing chloramphenicol (12.5 μg/ml), incubated at 37°C for 20 h. Since only the cDNA fragments with both *sfi*I A and *sfi*I B ends were allowed to be ligated into vector pDNR-LIB, only the recombinant clones were able to grow and were clearly identified as white colonies. The cDNA clones were randomly sampled and mini-prepared for a quality check using *Hin*dIII and *Eco*RI double-digestion to release inserts. The ligations with insert size larger than 500 bp were selected for large scale transformation. These colonies were subsequently picked and arrayed with a Q-Bot (Genetix, UK) into 384-well micro-titer plates. Each well on the culture plate contained 75 μl of LB freezing storage medium [360 mM K_2_HPO_4_, 132 mM KH_2_PO_4_, 17 mM Na citrate, 4 mM MgSO_4_, 68 mM (NH_4_)_2_SO_4_, 44% (v/v) glycerol, 12.5 μg/ml of chloramphenicol, LB]. Colonies were incubated at 37°C overnight, and then stored at -70°C.

### Full-length cDNA library evaluation and cDNA clone sequence analysis

To evaluate the quality of the current full-length cDNA library, 400 individual cDNA clones were randomly picked from 12 storage plates, and grown in 5 ml of LB with 12.5 μg/ml of chloramphenicol under 37°C with 200 rpm shaking for 16 h. Plasmid DNA was isolated using the alkaline-lysis method [30] and digested with *Hin*dIII and *Eco*RI. The cDNA inserts were analyzed by 1% agarose gel electrophoresis with ethidium bromide staining. The average cDNA insert size and the cDNA length distribution profiles were obtained.

Two hundred cDNA clones were randomly selected for sequencing analysis. Prior to sequencing, all plasmids were isolated from cDNA bacterial clones by cellular lysis and purified in 96-well plates. Single pass sequencing was performed from both directions using two "in-house" sequencing primers. Phred software [16] was used for base calling. Each sequence was edited manually by removing vector sequences and the ambiguous reads. The overlapping sequences (from both 3' and 5' ends) were evaluated and aligned into full consensus sequence contigs using the DNA analyzing software DNA for Windows 2.2.1 [12]. The non-overlapping sequences were formatted and treated as two separated sequence contigs. All aligned sequence contigs were analyzed with the Lasergene 5.0 software (DNA STAR, Madison, WI, USA) for identifying ORFs. Consensus sequences were searched against the National Center for Biotechnology Information (NCBI) [28] fungal database and the all-organism database under E-value of 10^-3 ^and 10^-6^, respectively. The genuine ORF fragments were cross validated by these two different scales of NCBI blast analysis.

## Authors' contributions

PL constructed the full-length cDNA library, participated in the cDNA sequencing and analysis, and drafted the manuscript; MW contributed to cDNA sequencing, Blast-searching the databases, and drafted the manuscript; XC conceived and coordinated the study, contributed materials and resources, interpreted the data, and wrote the manuscript; KGC contributed resources and participated in planning the experiemnets. All authors read and approved the final manuscript.
